# *Phyllanthus emblica* Prevents Adipogenesis by Regulating Histone Acetylation

**DOI:** 10.3390/foods14020160

**Published:** 2025-01-07

**Authors:** Seon Kyeong Park, Yu Geon Lee, Jae-In Lee, Min-Sun Kim, Jae-Ho Park, Jin-Taek Hwang, Min-Yu Chung

**Affiliations:** 1 Food Functionality Research Division, Korea Food Research Institute (KFRI), Wanju-gun 55365, Republic of Korea; p.seonkyeong@kfri.re.kr (S.K.P.); ugun2@kfri.re.kr (Y.G.L.); jaeinlee@kfri.re.kr (J.-I.L.); jaehopark@kfri.re.kr (J.-H.P.); jthwang@kfri.re.kr (J.-T.H.); 2 Food Industry Research Division, Korea Food Research Institute (KFRI), Wanju-gun 55365, Republic of Korea; mskim@kfri.re.kr; 3 Department of Food and Nutrition, Gangseo University, Seoul 07661, Republic of Korea

**Keywords:** *Phyllanthus emblica*, adipogenesis, gallic acid, histone acetylation, H3K9ac

## Abstract

*Phyllanthus emblica* is widely used in Ayurvedic preparations against multiple disorders and contains various bioactive components. This study aimed to determine the preventive effect of *P. emblica* on obesity by evaluating the inhibition of adipogenesis and the related regulatory epigenetic mechanisms during 3T3-L1 differentiation. The ethyl acetate fraction of *P. emblica* (EFPE) effectively inhibited lipid accumulation and triglyceride (TG) production in 3T3-L1 adipocytes. It also inhibited histone acetyltransferase (HAT) activity and regulated Pcaf-specific H3K9 acetylation and the expression of adipogenesis-related genes during adipocyte differentiation. Phenolic compounds were the main components of EFPE, of which gallic acid (GA) exhibited the strongest inhibitory effect on lipid accumulation and TG production. Notably, GA effectively regulated adipogenesis-mediated gene expression through H3K9 acetylation. These findings, along with the experiment results, suggest that EFPE containing GA is a potent agent for preventing obesity by regulating H3K9 acetylation.

## 1. Introduction

Obesity is characterized by the excessive accumulation of fat, in the form of triglycerides (TGs), in the adipose tissues due to energy-rich eating habits. It is associated with various metabolic disorders, including type 2 diabetes, cardiovascular disease, hypertension, and non-alcoholic fatty liver disease [1,2]. Adipogenesis plays a crucial role in energy storage within the body and is closely related to the development of obesity [3]. This complex process is regulated by adipogenic and lipogenic factors and leads to changes in cell morphology and gene expression patterns [4]. The key regulators, such as peroxisome proliferator-activated receptor gamma (PPAR-γ), CCAAT/enhancer binding proteins (C/EBPs), and sterol regulatory element-binding protein (SREBP-1), initiate adipocyte differentiation. Subsequently, the expression of lipogenic factors, including acetyl-CoA carboxylase (ACC) and fatty acid synthase (FASN), induces fatty acid synthesis and lipid accumulation [5]. Hence, a deeper understanding of the molecular mechanisms involved in adipogenesis is essential for developing strategies to prevent obesity.

Epigenetic regulation, such as DNA methylation, histone acetylation, and chromatin folding, is closely associated with gene activation and expression [6,7]. Histone acetylation affects the activation of transcription factors by modifying the chromatin structure around their target genes, thereby promoting their function by the addition of acetyl groups (-CH_3_CO) to the lysine residues in histone proteins [8]. In particular, histone acetyltransferases (HATs) and histone deacetylases (HDACs) play an important role in adipocyte differentiation and lipid metabolism [9]. HATs play a critical role in the expression of key transcription factors involved in the promotion of adipogenesis, including PPAR-γ and C/EBP-α. Therefore, the regulation of histone acetylation offers potential for the prevention of obesity, leading to the expression of adipogenic and lipogenic factors. A recent study demonstrated that certain phytochemicals affect epigenetic regulation by modulating the activity of HATs, which, in turn, has an effect on the development of obesity and other metabolic disorders [10]. Therefore, dietary compounds might hold promise as novel therapeutic agents for obesity prevention by the regulation of epigenetic changes [11].

*Phyllanthus emblica* L. is a widely distributed plant in subtropical and tropical regions, including China, India, Indonesia, and Malaysia [12]. The edible fruit of *P. emblica* is rich in vitamin C, fatty acids, tannins, alkaloids, ellagitannins, gallic acid (GA), emblicanin A, emblicanin B, rutin, and quercetin [13,14]. *P. emblica*, which contains a variety of bioactive components, has been widely used in Ayurvedic herbal preparations for treating diabetes. It has been shown to have hypolipidemic and anti-aging properties and is also used as a diuretic, aphrodisiac, and UV protectant [2,13]. Therefore, this study aimed to assess the role of *P. emblica* fractions and their major components in obesity prevention through the regulation of adipogenesis via histone acetylation.

## 2. Materials and Methods

### 2.1. Sample Preparation

*P. emblica* powder was extracted using 70% ethanol at 40 °C for 2 h in a reflux condenser. The extracted *P. emblica* was filtered through Whatman No. 2 filter paper (Whatman International Limited, Kent, UK), and concentrated using a rotary vacuum evaporator (N-1000; Eyela Co., Tokyo, Japan). To separate the active compounds of *P. emblica*, the concentrated EtOH extract was consecutively fractionated using five solvents, namely n-hexane, chloroform, ethyl acetate (EtOAc), n-butanol, and distilled water, in a separating funnel. Each solvent fraction was concentrated, lyophilized using a vacuum-tray freeze dryer (Operon, Gimpo, Republic of Korea) for 3 days, and stored at −20 °C.

### 2.2. Histone Acetyltransferase (HAT) Activity Assay

Using the HeLa cell nuclear extract (ab286851, Abcam, Cambridge, MA, USA) as the enzyme source, the HAT inhibitory effect of *P. emblica* was measured using a commercially available kit (Histone Acetyltransferase activity assay kit, ab65352, Abcam, Cambridge, UK), according to the manufacturer’s instructions.

### 2.3. Cell Culture and Differentiation

3T3-L1 preadipocytes were obtained from the American Type Culture Collection (ATCC, Manassas, VA, USA). And preadipocytes were cultured in high-glucose Dulbecco’s modified Eagle’s medium (DMEM) supplemented with 10% bovine calf serum (BCA) and 1% antibiotics (Welgene, Daegu, Republic of Korea) in a humidified atmosphere of 5% CO_2_ at 37 °C in an incubator. 3T3-L1 preadipocytes were continuously maintained to ensure that their confluence did not exceed 80%.

To induce 3T3-L1 differentiation, the 3T3-L1 preadipocytes were seeded at a density of 1.5 × 10^5^ cells/well density on a 6-well plate and cultured until 100% confluence was reached. Differentiation was also induced using DMEM supplemented with 10% fetal bovine serum (FBS-DMEM). The confluent cells (Day 0) were treated with a mixture of 0.5 mM 3-isobutyl-1-methylxathine, 1 μM dexamethasone, and 10 μg/mL insulin (MDI) in FBS-DMEM, with samples dissolved in DMSO (final conc. 0.1% DMSO). On day 3, the medium of the differentiated 3T3-L1 cells was replaced with insulin media (10 μg/mL insulin in FBS-DMEM) and samples or DMSO, followed by incubation for 48 h. On days 5 and 7, the culture medium was replaced again with insulin media and samples or DMSO every 2 days. During 3T3-L1 differentiation, FBS-DMEM was treated with 0.1% DMSO in normal control (NC).

### 2.4. Cell Viability

Confluent 3T3-L1 preadipocytes were treated with the samples or 0.1% DMSO for 48 h. Following treatment, 3-(4,5-dimethyl-2-thiazolyl)-2,5-diphenyltetrazolium bromide (MTT) stock solution (Sigma, St. Louis, MO, USA) was added to each well. After 2 h, the MTT formazans were dissolved in DMSO, and the absorbance was measured at 570 nm (measured wave) and 660 nm (reference wave) using a microplate reader (Molecular Devices, Sunnyvale, CA, USA) to determine cell viability.

### 2.5. Oil-Red-O (ORO) Staining Assay

Intracellular lipid accumulation in differentiated 3T3-L1 adipocytes was assessed using an ORO staining assay. Briefly, the differentiated 3T3-L1 cells were washed with ice-cold Dulbecco’s phosphate-buffered saline (DPBS) and fixed with 3.7% paraformaldehyde for 20 min. The fixed cells were washed with 60% isopropyl alcohol (IPA) and stained with ORO dye solution for 20 min. The stained intracellular lipid droplets were rinsed thrice with distilled water. The stained dye was dissolved using IPA and transferred to a 96-well plate. Its absorbance was subsequently measured at 490 nm using a microplate reader (Molecular Devices, Sunnyvale, CA, USA).

### 2.6. Triglyceride (TG) Content

To evaluate the TG content, the adipocytes were collected in 5% NP-40 buffer and heated at 100 °C for 5 min, until the solution became opaque. They were subsequently cooled to room temperature, followed by centrifugation at 13,000× *g* and 2 min. The TG content of the supernatant was measured using a commercially available kit (EZ-Triglyceride Quantification Assay kit, DoGenBio Co., Ltd., Seoul, Republic of Korea), according to the manufacturer’s instructions. Briefly, lipase was added to the diluted supernatant, followed by incubation for 20 min. After the addition of the reaction mixture, the supernatant was incubated for 30 min, and the O.D. was measured at 570 nm using a microplate reader (Molecular Devices, Sunnyvale, CA, USA).

### 2.7. Extraction of Histone from 3T3-L1 Cells

Histone was isolated using the histone extraction kit (ab113476, Abcam, Cambridge, MA, USA), according to the manufacturer’s protocol. Briefly, the 3T3-L1 cells harvested with DPBS were centrifuged at 300× *g* for 5 min at 4 °C. The cell pellets were resuspended in 1× pre-lysis buffer and incubated on ice for 10 min with gentle stirring. Thereafter, the resuspended cells were centrifuged at 10,000× *g* for 1 min at 4 °C, and cell pellets were suspended in lysis buffer for 30 min on ice. The lysed cells were centrifuged at 13,000× *g* for 5 min at 4 °C, and the supernatants were transferred into a new tube with 0.3 volume Balance-DTT solution.

### 2.8. Quantitative Real-Time RT-PCR

To isolate total RNA, differentiated 3T3-L1 adipocytes were harvested using RNAiso Plus (TaKaRa, Shiga, Japan). And cDNA synthesis was performed using the ReverTra Ace qPCR RT Master Mix with a gDNA remover kit (Toyobo, Osaka, Japan), according to the manufacturer’s guidelines. Briefly, the cDNA was synthesized from 2 μg of RNA, followed by qRT-PCR using the iCycle iQ system (Bio-Rad, Hercules, CA, USA) and SYBR Green Master Mix (Roche, Basel, Switzerland). The primers used for qRT-PCR assays are provided in Supplementary Table S1. Expression levels were normalized to β-actin and quantified using the comparative method.

### 2.9. Western Blotting

To determine protein expression, mature 3T3-L1 adipocytes were harvested using a lysis buffer (Cell Signaling Technology, Beverly, MA, USA) containing phosphatase and protease inhibitors on ice. Briefly, the cell lysates were centrifuged at 13,000× *g* for 20 min at 4 °C, and the protein was quantified using the BSA assay. Proteins were separated using Mini-PROTEAN TGX Gels (Bio-Rad) and transferred onto PVDF membranes (Bio-Rad). The transferred proteins were blocked in EverBlot Blocking buffer (Bio-Rad), and the membranes were incubated with each primary antibody at 4 °C with shaking. After incubation, the membrane was incubated with secondary antibodies at RT for 2 h. The antibodies used are listed in Supplementary Table S2.

### 2.10. Analysis of Main Compounds Using LC-MS/MS

The major components of the extracts were analyzed using a SCIEX Exion LC coupled with Q-TOF-MS (X500R, AB SCIEX, Concord, ON, Canada). The different compounds were separated using a Hypersil GOLD™ VANQUISH™ C18 UHPLC column (2.1 mm × 150 mm, 1.9 µm), which was maintained at a flow rate of 0.45 mL/min at 40 °C. The mobile phases were composed of solvent A (0.1% [*v*/*v*] formic acid in water) and solvent B (0.1% [*v*/*v*] formic acid in acetonitrile), and gradient conditions were conducted as follows: a linear increase of 1–5% B at 0–1 min, 5–25% B at 1–3 min, 25–35% B at 3–4.8 min, 25–35% B at 4.8–5.8 min, 35–45% B at 6.8–7.8 min, 45–60% B at 8.8–9.3 min, and 60–100% B at 9.3–10 min. The MS conditions were as follows: mass range 50–1100 *m*/*z*, collision energy −10 V, ion spray voltage −4500 V, gas temperature 500 °C, decluttering potential −90 V, curtain gas 30 psi, nebulizing gas 50 psi, and drying gas 60 psi. Data analysis was performed using SCIEX OS 1.6.2. (SCIEX, Framingham, MA, USA). The MS/MS analysis was performed using the information-dependent acquisition (IDA) scanning at −30 eV collision energy. Quantitative analysis of GA and galloylquinic acid (GQA) in *P. emblica* was performed using the SCIEX Exion LC system under the same LC analysis conditions. Calculations were conducted by comparing the retention times and peak areas of the samples with those of the standard compounds.

### 2.11. Statistical Analysis

Results are presented as the mean ± SD, and statistical significances were considered at *p* < 0.05. Analyses were performed using Prism 8.1 (GraphPad Software, San Diego, CA, USA) with one-way analysis of variance (ANOVA) with Tukey’s multiple comparison test.

## 3. Results

### 3.1. Inhibitory Effect of P. emblica on Lipid Accumulation in 3T3-L1 Adipocytes

**Figure 1:**
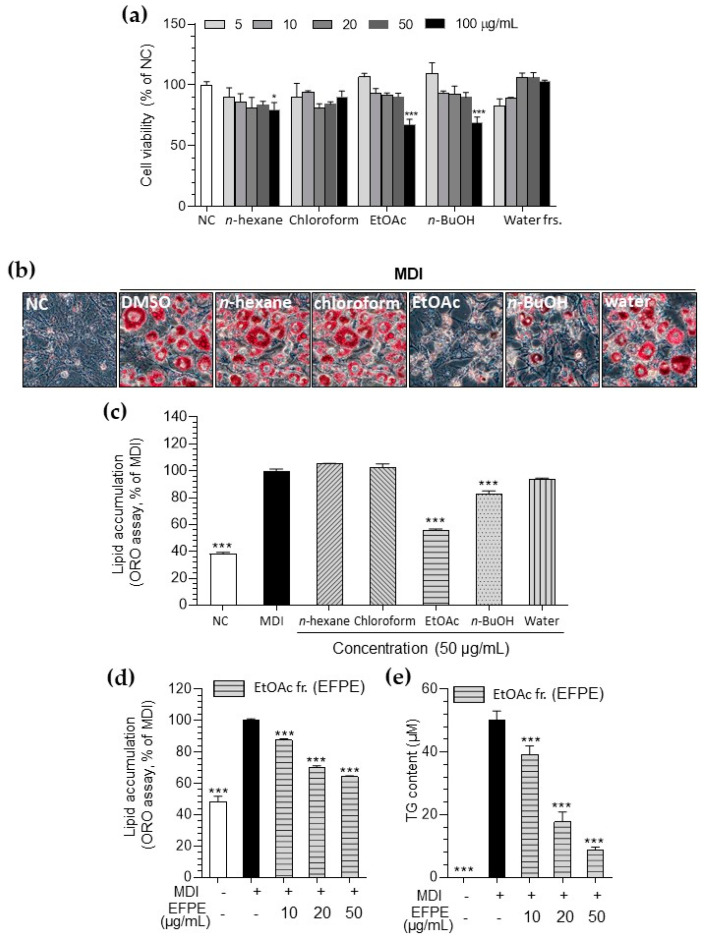
Effect of *P. emblica* fractions on viability (**a**), the corresponding images (**b**), and lipid accumulation content (**c**) in 3T3−L1 cells. The inhibitory effect of ethyl acetate fraction from *P. emblica* (EFPE) on lipid accumulation (**d**) and triglyceride (TG) content (**e**) in 3T3−L1 adipocytes. The results are expressed as the mean ± SD from three independent experiments, each performed with technical triplicates. Results were statistically analyzed by performing a one-way ANOVA test, followed by Dunnet’s multiple comparison test comparing all other groups with the MDI group (*** *p* < 0.001, * *p* < 0.05).

Since the chloroform, ethyl acetate, and butanol fractions showed cytotoxicity above a concentration of 100 μg/mL in 3T3-L1 cells (Figure 1a), the experiments were performed at concentrations below 50 μg/mL. The ethyl acetate fraction from *P. emblica* (EFPE; 78.01%) showed the highest inhibitory effect on lipid accumulation in 3T3-L1 adipocytes compared to other fractions at 50 μg/mL concentration (Figure 1b,c). EFPE treatment effectively blocked TG production and total lipid accumulation in a concentration-dependent manner (Figure 1d,e).

### 3.2. Inhibitory Effect of Various Fractions of P. emblica Against HAT Activity in a Cell-Free System

**Figure 2:**
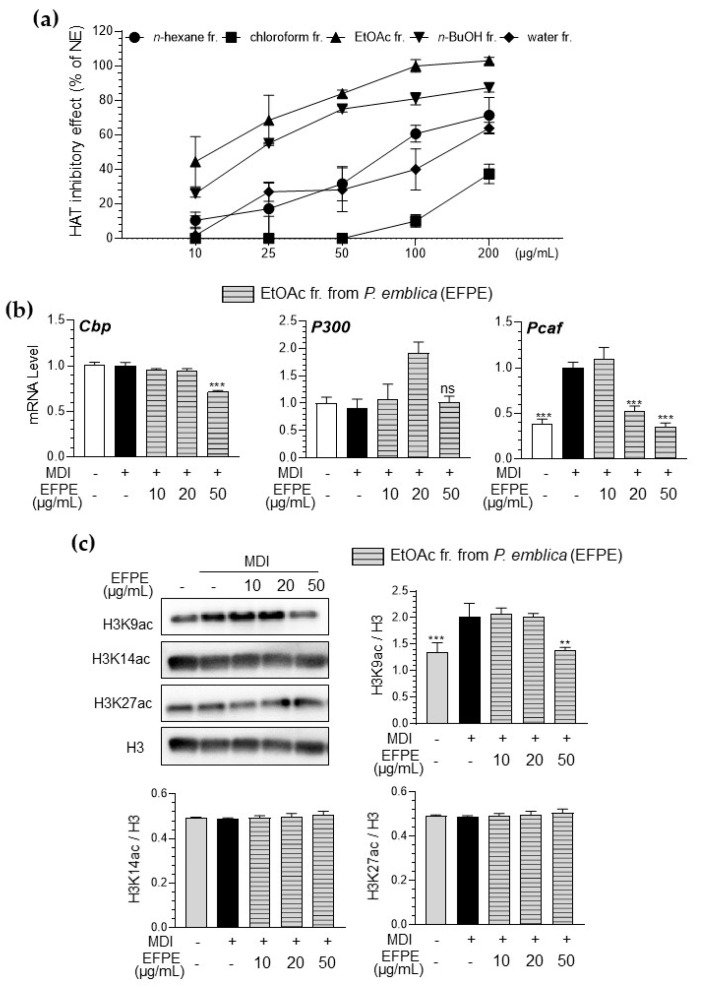
Inhibitory effect of *P. emblica* fractions on histone acetyltransferase (HAT) activity in a cell-free system (**a**). Effect of ethyl acetate fraction from *P. emblica* (EFPE) on HAT-specific mRNA expression (Cbp, P300, and Pcaf) (**b**), and histone acetylation (H3K9ac, H3K14ac, and H3K27ac) protein level (**c**) in 3T3−L1 adipocytes. The results are expressed as the mean ± SD from three independent experiments, each performed with technical triplicates. Results were statistically analyzed by performing a one−way ANOVA test, followed by Dunnet’s multiple comparison test comparing all other groups with the MDI group (*** *p* < 0.001, ** *p* < 0.01, ns: not significant (*p* > 0.05)).

EFPE (IC_50_ = 13.43 μg/mL) showed the highest inhibitory effect against HAT activity compared with other fractions from *P. emblica* (IC_50_ of n-hexane; 81.48 μg/mL, chloroform; not detected, n-butanol; 22.34 μg/mL, and distilled water fractions; 162.37 μg/mL) (Figure 2a).

The effect of EFPE on histone acetylation in differentiated 3T3-L1 adipocytes was also evaluated by measuring the expression levels of P300, Cbp, and p300/CBP-associated factor (Pcaf) as well as the acetylation levels of histone proteins. As shown in Figure 2b, MDI-induced 3T3-L1 adipocytes showed increased Pcaf gene and protein expression, whereas EFPE effectively inhibited Pcaf mRNA expression. The level of acetylation at the lysine residues of histone proteins, particularly H3K9 residues, was significantly increased in 3T3-L1 adipocytes, whereas it was effectively attenuated by treatment with EFPE (Figure 2c).

### 3.3. EFPE Regulates Adipogenesis-Related Gene Expression in 3T3-L1 Adipocytes

**Figure 3:**
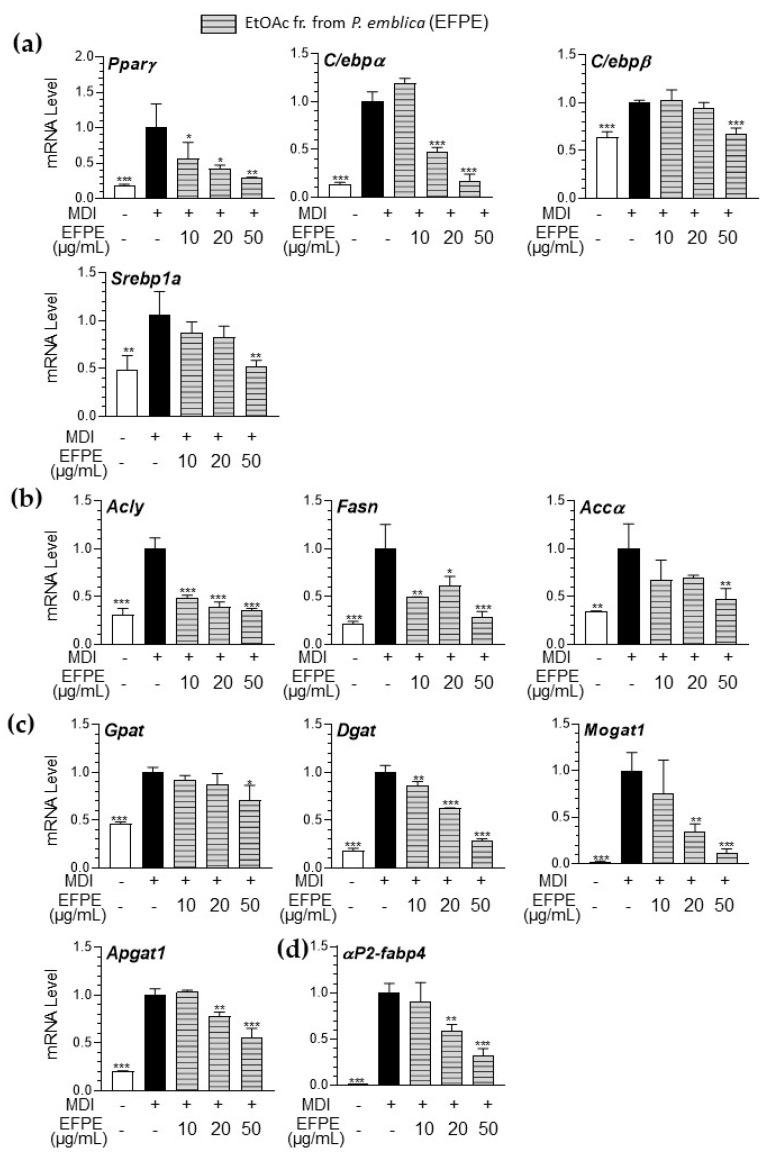
Effect of ethyl acetate fraction from *P. emblica* (EFPE) on adipogenesis-related mRNA expression, including transcription factors (**a**), fatty acid synthesis (**b**), TG synthesis (**c**), and lipid transport (**d**) in 3T3−L1 adipocytes. The results are expressed as the mean ± SD from three independent experiments, each performed with technical triplicates. Results were statistically analyzed by performing a one-way ANOVA test, followed by Dunnet’s multiple comparison test comparing all other groups with the MDI group (*** *p* < 0.001, ** *p* < 0.01, * *p* < 0.05).

To evaluate the regulatory effect of EFPE on the mechanisms controlling lipid accumulation in 3T3-L1 adipocytes, we assessed the expression of genes involved in metabolism associated with adipogenesis, including transcription factors (Ppar-γ, C/ebp-α/β, and Srebp-1α), fatty acid synthesis genes (Acly, Fasn, and Accα), TG synthesis genes (glycerol-3-phosphate acyltransferase; Gpat, diacylglycerol acyltransferase; Dgat, monoacylglycerol acyltransferase; Mogat1, and 1-acylglycerol-3-phosphate O-acyltransferase 2; Apgat1), and lipid transport genes (αP2-fabp4). The mRNA expression of genes associated with adipogenesis increased during MDI-induced 3T3-L1 differentiation and was significantly downregulated by treatment with EFPE (Figure 3).

### 3.4. Analysis of Bioactive Components in EFPE

**Figure 4:**
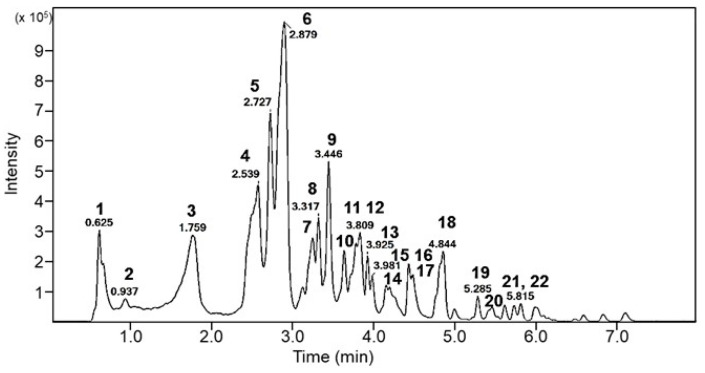
UPLC chromatogram of ethyl acetate fraction from *P. emblica* (EFPE).

Phytochemicals in EFPE were analyzed using LC Q-TOF/MS analysis and IDA mode MS/MS scanning. Compounds were identified by comparing the MS/MS fragmentation with those reported previously (Figure 4 and Table 1). One compound showed the same precursor ions (peaks 3, 5 and 6; *m*/*z* 687) in the negative-ion MS scan, and its MS/MS fragment ions were observed at *m*/*z* 343, 191, 169, 147, and 85. This compound was identified as the isomer of GQA containing GA (*m*/*z* 169) and quinic acid (*m*/*z* 191). Another main compound identified was GA (*m*/*z* 169) with two fragment ions (*m*/*z* 125, 98). Interestingly, GA (*m*/*z* 169) was commonly detected in the major fragment ions of other compounds, such as peaks 7, 8, 9, 11, 12, 13, 16, 17, 20, 21, and 22. Therefore, the main compounds of the EFPE were confirmed to be phenolic compounds such as GA, GQA, and GA derivatives. Notably, the quantitative analysis revealed that EFPE contained 13.99 and 10.71 mg/g of GA and GQA, respectively (Table 2).

### 3.5. Inhibitory Effect of GA and GQA on Lipid Accumulation in 3T3-L1 Adipocytes

**Figure 5:**
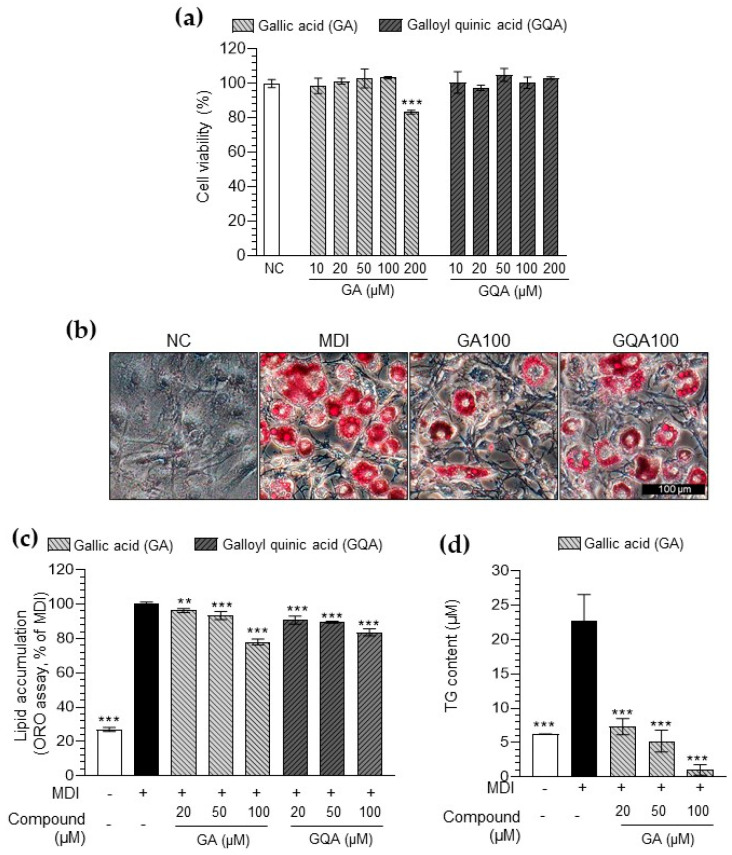
Effect of gallic acid (GA) and galloylquinic acid (GQA) on viability (**a**), the corresponding images (**b**), lipid accumulation content (**c**), and triglyceride (TG) content (**d**) in 3T3−L1 adipocytes. The results are expressed as the mean ± SD from three independent experiments, each performed with technical triplicates. Results were statistically analyzed by performing a one−way ANOVA test, followed by Dunnet’s multiple comparison test comparing all other groups with the MDI group (*** *p* < 0.001, ** *p* < 0.01).

Both GA and GQA did not show toxicity at concentrations below 100 μM (Figure 5a). Hence, subsequent experiments were performed at concentrations of 100 μM or less. GA and GQA significantly decreased lipid accumulation in 3T3-L1 adipocytes (Figure 5b,c). Notably, GA showed more significant inhibition of lipid accumulation than GQA at 100 μM. Furthermore, GA suppressed TG production in the 3T3-L1 adipocytes (Figure 5d). These results confirmed the effectiveness of GA against lipid accumulation.

### 3.6. Inhibitory Effect of GA and GQA on HAT Activity and Histone Acetylation

**Figure 6:**
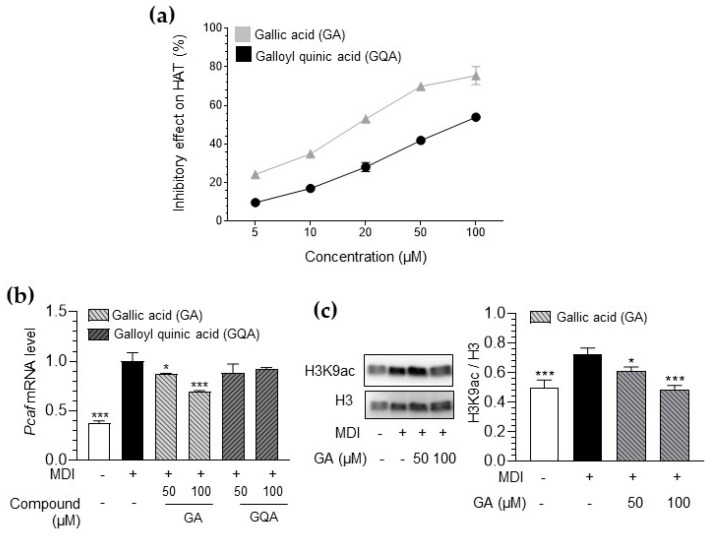
Inhibitory effect of gallic acid (GA) and galloylquinic acid (GQA) on histone acetyltransferase (HAT) activity in a cell-free system (**a**). Effect of GA and GQA on Pcaf mRNA expression (**b**) and H3K9ac protein level (**c**) in 3T3−L1 adipocytes. The results are expressed as the mean ± SD from three independent experiments, each performed with technical triplicates. Results were statistically analyzed by performing a one−way ANOVA test, followed by Dunnet’s multiple comparison test comparing all other groups with the MDI group (*** *p* < 0.001, * *p* < 0.05).

To confirm the physiologically active compounds of EFPE, a comparative evaluation of the inhibitory effects of GA and GQA on histone acetylation was performed. First, compared with GQA, GA effectively inhibited HAT activity in a cell-free system (Figure 6a and Table 3). The IC_50_ value of GA (IC_50_ = 18.32 μM) was approximately 4.6 times lower than that of GQA (IC_50_ = 83.65 μM).

Additionally, GA treatment significantly reduced the elevated levels of Pcaf mRNA in 3T3-L1 adipocytes (Figure 6b). In contrast, GQA treatment did not inhibit Pcaf mRNA levels in 3T3-L1 adipocytes. Furthermore, GA effectively blocked the acetylation of H3K9 residues in these cells (Figure 6c). These results suggest that the inhibitory effect of EFPE on histone acetylation is due to the action of the bioactive compound GA.

### 3.7. GA and GQA Regulates Adipogenesis-Related Gene Expression in 3T3-L1 Adipocytes

**Figure 7:**
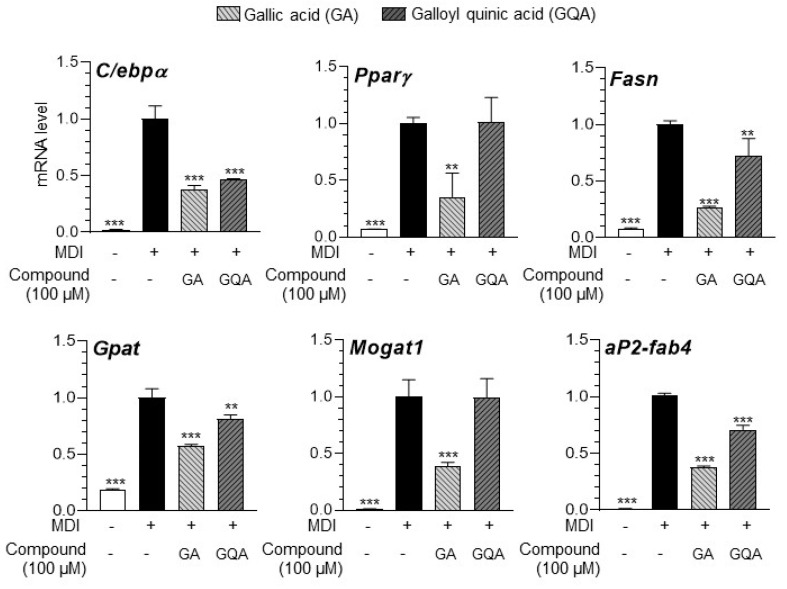
Inhibitory effect of gallic acid (GA) and galloylquinic acid (GQA) on adipogenesis-related mRNA (C/ebpα, Pparγ, Fasn, Gpat, Mogat1, and αP2-fab2) expression. The results are expressed as the mean ± SD from three independent experiments, each performed with technical triplicates. Results were statistically analyzed by performing a one-way ANOVA test, followed by Dunnet’s multiple comparison test comparing all other groups with the MDI group (*** *p* < 0.001, ** *p* < 0.01).

The regulatory effect of the compounds on adipogenesis-associated gene expression in 3T3-L1 adipocytes was assessed using qRT-PCR. Both GA and GQA downregulated the expression of genes associated with lipid accumulation (Figure 7). However, the effect of GA on the mRNA expression of transcription factors (C/ebp-α and Ppar-γ), fatty acid synthesis genes (Fasn), TG synthesis genes (Gpat and Mogat1), and lipid transport genes (αP2-fabp4) was higher than GQA.

## 4. Discussion

Obesity arises from the accumulation of excess energy in the body, primarily stored as adipose tissue [15]. During adipocyte differentiation, the expression of adipogenic genes is regulated by various transcription factors and specific regulatory regions [16], highlighting the importance of epigenetic regulations, including histone modification, DNA methylation, and chromatin remodeling, in regulating adipocyte differentiation. Lifestyle and diet influence epigenetic changes, and dietary factors and their metabolites affect epigenetic mechanisms, thereby contributing to health benefits [16,17]. In this experiment, we evaluated the significance of *P. emblica* as a food source in the regulation of obesity through epigenetic action.

Adipogenesis involves two major processes: preadipocyte proliferation and adipocyte differentiation [3]. It was observed that specific concentrations of *P. emblica* fractions had an effect on preadipocyte proliferation (Figure 1). Interestingly, EFPE, which demonstrated the strongest inhibition of lipid accumulation, was found to have no impact on cell proliferation at concentrations of 10–50 μg/mL. Based on this understanding, the inhibitory effect of EFPE on lipid accumulation is presumed to be due to the regulation of adipocyte differentiation rather than preadipocyte proliferation. Furthermore, a previous report suggested that *P. emblica* extract exerts anti-obesity effects by regulating apoptosis-mediated mechanisms in 3T3-L1 adipocytes [2]. Animal studies have also reported the potential anti-obesity effect of *P. emblica* [18,19]. *P. emblica* exhibited hepatoprotective effects in a Sprague Dawley rat model of high-fat-diet-induced non-alcoholic fatty liver disease [19]. Chen et al. (2023) [18] reported that *P. emblica* supplementation ameliorated cognitive dysfunction through weight loss and gut microbiota modulation in rats. Although various studies have reported the anti-obesity effects of *P. emblica*, its underlying mechanisms remain unclear. In the experimental results, we evaluated the effects of *P. emblica* on histone acetylation during adipocyte differentiation.

Core histone proteins influence the regulation of gene transcription by controlling the accessibility of transcription factors to target genes [20]. Notably, histone acetylation and HAT activity are closely associated with adipocyte differentiation [21,22]. Some studies have suggested that food-derived components may prevent obesity by inhibiting HAT activity. Extracts of *Capsella bursa-pastori*, *Plumbago rosea* root, and grape seed have been shown to inhibit HAT activity [20,21,22]. In particular, the *Quercus acutissima* fruit extract has been shown to have an anti-obesity effect with HAT inhibition in 3T3-L1 cells and retroperitoneal fat in high-fat-diet-induced obese mice [23]. As shown in Figure 2, the fractions from *P. emblica* effectively inhibited HAT activity. In particular, the EFPE showed the highest HAT inhibitory effect, which was similar to the results of its inhibitory effects on lipid accumulation. EFPE, as a HAT inhibitor, suppressed Pcaf expression and H3K9 acetylation (Figure 2). Acetylation of histone lysine residues is correlated with gene activation and is catalyzed by site-specific HAT, which is the reversible acetylation of N-terminal lysine residues at positions 9, 14, 18, and 23 of H3 and positions 5, 8, 12, and 16 of H4 [22]. This process results in decondensation of the nucleosome structure, modification of histone and DNA interactions, and binding of transcription factors [24]. Among HATs, CBP/p300 is associated with nuclear receptor-mediated H3K18/27ac, and GCN5/PCAF plays an important role in H3K9ac [25]. These findings emphasize the unique substrate- and site-specificity of HATs within cells while also demonstrating the distinct roles of GCN5/PCAF- and CBP/p300-mediated histone acetylation in gene activation [25]. In particular, GCN5 and PCAF increase the expression of adipogenesis-related genes through the acetylation of N-terminal lysine residues at position 9 of H3 (H3K9ac) [26]. Based on these findings, it is confirmed that EFPE is an effective dietary HAT inhibitor showing H3K9ac inhibition activity during 3T3-L1 differentiation.

Three transcription factors (PPARγ, C/EBPs, and SREBPs) play critical roles in the early stages of adipogenesis, and adipocyte maturation activates the expression of metabolic genes through a transcriptional cascade reaction [21]. The C/EBPs and the basic helix-loop-helix protein ADD-1/SREBP-1 are induced during adipocyte differentiation, and they accelerate this process by regulating the expression of PPARγ and providing ligands for this receptor [3]. PPARγ and C/EBPα also play pivotal roles in the final step of adipocyte differentiation. SREBP-1 stimulates the transcription of genes associated with lipogenesis, including Fasn, thereby promoting fatty acid production [27]. In diet-induced obesity models, acetylation of H3K9/H3K18 has been identified as a potential prognostic marker for predicting metabolic syndromes and hepatic steatosis in obesity [28]. HAT activity is closely related to adipogenesis-related gene expression in fat cells [25]. Moreover, HAT activation and histone acetylation (H3K9, H4K8, H4K16) lead to the upregulation of adipogenic genes, including PPARγ, ACLY, and FASN, during fatty acid-induced lipid accumulation [29]. Notably, PCAF is abundantly expressed during adipocyte differentiation and has been shown to function upstream of PPARs and C/EBP during adipogenesis [30,31]. In 3T3-L1 adipocytes, Pcaf knockout impairs the differentiation process by inhibiting the expression of adipogenic transcription factors, including C/EBPα and PPARγ [31]. Based on these findings, our study suggests that the regulation of PCAF-related histone acetylation by EFPE results in a decrease in adipogenic gene expression.

LC-QTOF/MS analysis was performed to confirm the main component of EFPE. The main components of the EFPE were phenolic compounds with gallic acid as the basic structure, including GA and GQA (Figure 4 and Table 1). Both GA and GQA effectively inhibited lipid accumulation through the regulation of histone acetylation (Figure 5 and Figure 6). However, GA showed more effective regulation of HAT inhibition and adipogenesis when compared with GQA.

Nutrients function as regulators of DNA methylation and histone modification, either by directly inhibiting the enzymes involved in these processes or by modulating the availability of substrates required for enzymatic activity [24]. Recent studies suggest that phytochemicals, including butyrate, sulforaphane, and curcumin, may exert their health benefits through epigenetic modifications such as HAT and HDAC activity [17]. GA (3, 4, 5-trihydroxy benzoic acid) is a natural endogenous phenolic acid [32] and the basic component of various anti-obesity agents, including epigallocatechin gallate, ethyl gallate, gallocatechin gallate, methyl gallate, propyl gallate, and theaflavin-3-gallate [33]. The proposed mechanism underlying the anti-obesity effects of GA involves a reduction in adipocyte size and number through the induction of apoptosis in 3T3-L1 cells [33]. However, the potential anti-obesity effects of GA mediated via epigenetic regulation during adipocyte differentiation remain unexplored. Several studies have reported that GA is a strong HAT inhibitor [24,34]. GA effectively inhibited NF-κB signaling by P300/CBP-dependent HAT inhibition on LPS-induced inflammation in A549 cells [34]. GA has also been shown to ameliorate cognitive dysfunction and inflammatory responses through HAT inhibition in Aβ-induced neuroinflammation. A previous study demonstrated that Aβ-mediated NF-κB activation involves Pcaf rather than p300/Cbp, and GA was shown to effectively inhibit Pcaf [35]. The results of this study also showed that GA effectively inhibited Pcaf-mediated histone acetylation during adipocyte differentiation.

The role of GQA as HAT inhibitor has not yet been reported. Bora-Tatar et al. (2009) [36] reported that the epigenetic regulatory effects of caffeoylquinic acid, including DNA methylation and histone acetylation, were attributed to caffeic acid, underscoring that these effects are not mediated by quinic acid. Similarly, GQA exhibited relatively lower HAT inhibition compared to GA and confirmed lower anti-adipogenic effects during adipocyte differentiation. These findings suggest that the impact of GQA is likely attributed to the gallic acid.

Based on these results, *P. emblica*, which contains the HAT inhibitor GA, was found to regulate adipogenesis through the modulation of histone acetylation. The findings of this study confirm that *P. emblica* exerts its effects on adipogenesis by regulating histone acetylation.

## 5. Conclusions

In this study, we focused on the anti-adipogenic effect of *P. emblica* and GA through the regulation of histone acetylation during adipocyte differentiation. EFPE and GA effectively prevented lipid accumulation in 3T3-L1 adipocytes. Furthermore, they also inhibited HATs in a cell-free system and Pcaf-mediated H3K9ac during adipocyte differentiation. As a result of the inhibition of histone acetylation, EFPE and GA led to the downregulation of key transcriptional factors during adipocyte differentiation. The anti-adipogenic effects of EFPE and GA are likely attributed to the inhibition of histone acetylation. The impact of *P. emblica* during adipocyte differentiation represents a significant potential for advancing the development of functional food ingredients targeting obesity management. However, further investigations are required to elucidate the specific bioactive constituents and their underlying mechanisms through animal and clinical studies. These further studies could provide definitive evidence supporting the potential of *P. emblica* as a functional food ingredient for obesity prevention and management.

## Figures and Tables

**Table 1:** List of identified components from the ethyl acetate fraction of *P. emblica* (EFPE) using UPLC-MS.

Peak No.	Rt (min)	Proposed Compound	Precursor (*m*/*z*) [M−H]^−^	MS/MS Fragments
1	0.63	Mucic acid gallate	361	209, 191, 147, 133, 85, 71, 59
2	0.94	Mucic acid gallate	723.1 [2M−H]^−^	361, 209, 191
3	1.76	Galloylquinic acid isomer	687.1	343, 191, 147, 85
4	2.54	Gallic acid	169	169, 125, 124, 123, 107, 97, 95, 81, 79, 69, 67, 53
5	2.73	Galloylquinic acid isomer	687.1	687, 343, 191, 169, 147, 85
6	2.88	Galloylquinic acid isomer	687.1	687, 343, 237, 191, 169, 147, 85
7	3.12	Gallic acid derivative	673.1	387, 285, 169, 133, 125, 115
8	3.24	Gallic acid derivative	387.1	169, 125, 129
9	3.32	Gallic acid derivative	455.0 (387)	387, 325, 209, 191, 173, 169, 151, 129, 125
10	3.45	Digalloyl glucose	483.1	483, 331, 313, 271, 241, 211, 169, 168, 151, 125, 124
11	3.64	Digallic acid	321	169, 125
12	3.81	Trigalloyl glucose isomer	635.1	123, 113, 93, 68, 25
13	3.93	Trigalloyl glucose isomer	635.1	483, 465, 423, 313, 271, 211, 169
14	3.98	Digalloyl-HHDP glucose	785.1	633, 615, 300, 463, 275
15	4.16	unidentified ellagitannin	953.6	300
16	4.21	Gallic acid derivative	965.1	387, 285, 169, 133, 125
17	4.45	Gallic acid derivative	197	169, 125, 124, 78
17	4.84	Kaempferol-3-O-glucoside	447.1	479, 314, 287, 223, 213, 163
19	5.29	Kaempferol-3-O-deoxyhexoside	431.1	285, 284, 255, 229, 227
20	5.62	unidentified gallic acid	461.1	313, 271, 241, 211, 169, 151, 125, 124
21	5.73	unidentified gallic acid	461.1	313, 211, 189, 169, 161, 151, 147, 125, 124, 123
22	5.82		613.1	465, 461, 313, 271, 211, 169

**Table 2:** Quantitative analysis of gallic acid (GA) and galloylquinic acid (GQA).

Compound Name	Phenolic Acid (mg/g)
Gallic acid (GA)	13.99 ± 0.07
3-Galloylquinic acid (GQA)	10.71 ± 0.16

**Table 3:** Inhibitory effect against HAT activity on gallic acid (GA) and galloylquinic acid (GQA).

Compound Name	IC_50_ (μM)
Gallic acid (GA)	18.32
3-Galloylquinic acid (GQA)	83.65

## Data Availability

The original contributions presented in this study are included in the article/Supplementary Materials. Further inquiries can be directed to the corresponding author.

## Supplementary Materials

The following supporting information can be downloaded at: https://www.mdpi.com/article/10.3390/foods14020160/s1, Table S1. Primer sequences used for qRT-PCR assay; Table S2. Antibody list used for Western blot assay.
