# Supplementary material for: Phyllanthus emblica Prevents Adipogenesis by Regulating Histone Acetylation
Source: Foods. 2025 Jan 7;14(2):160. doi: 10.3390/foods14020160 (PMC11764537; doi:10.3390/foods14020160)
Supplement: Supplementary file 1 [file foods-14-00160-s001.zip › foods-3368582-supplementary.pdf]

Supplementary Table S1. Primer sequences used for qRT-PCR assay

Gene name	Sequence (5'-3')	
	Forward	Reverse
Cbp	GAC CGC TTT GTT TAT ACC TGC	TCT TAT GGG TGT GGC TCT TTG
P300	GTT GCT ATG GGA AAC AGT TAT GC	TGT AGT TTG AGG TTG GGA AGG
Pcaf	TGT CAT TGG TGG TAT CTG T	ATA TGT GAG GAA GTT GAG GAT
Gcn5	TGT CCA AAC CTA GCA ACG	GTC ATC ACT CAT GCG GAA
Pparγ	CCACCAACTTCGGAATCAGCT	TTTGTGGATCCGGCAGTTAAGA
C/ebpα	TAC CGA GTA GGG GGA GCA	TCA TTT TTC TCA CGG GGC C
C/ebpβ	AAG ATG CGC AAC CTG GAG A	TTG AAC AAG TTC CGC AGG G
Acly	ACA TTG CAG ACC TGG ATG CCA	TTA AGG AGG AAG TTG GCA GT
Fasn	AGAAGCCATGTGGGGAAGATT	AGCAGGGACAGGACAAGACAA
Accα	TCTATTCGGGGTGACTTTC	CTATCAGTCTGTCCAGCCC
Gpat	GTA GTT GAA CTC CTC CGA CA	ATC CAC TAC CAC TGA GAG GA
Dgat	GGC CCA AGG TAG AAG AGG AC	GAT CAG CAT CAC CAC ACA CC
Mogat1	CTG GTT CTG TTT CCC GTT GT	TGG GTC AAG GCC ATC TTA AC
Apgat1	AGC GGA CAG AAG AAA CTG GA	TGA AGT AGA CAC CCC CAA GG
αP2-fabp4	GGG AAC CTG GAA GCT TGT CT	ACT CTC TGA CCG GAT GGT GA
Actin	CTA AGG CCA ACC GTG AAA G	ACC AGA GGC ATA CAG GGA CA

Supplementary Table S2. Antibody list used for Western blot assay

Antibody	Species	Dilution	Company	Catalog
anti-H3K9ac	Rabbit	1:1000	Cell signaling	9649S
anti-H3K14ac	Rabbit	1:1000	Abcam	ab82501
anti-H3K27ac	Rabbit	1:1000	Abcam	ab177178
anti-Histone H3	Rabbit	1:500	Santa Cruz	sc-10809
Goat anti-Rabbit IgG (H+L) Secondary Antibody		1:10,000	Thermo Fisher Scientific Inc.	31460
